# Detection of Low Density Lipoprotein—Comparison of Electrochemical Immuno- and Aptasensor

**DOI:** 10.3390/s21227733

**Published:** 2021-11-20

**Authors:** Daria Rudewicz-Kowalczyk, Iwona Grabowska

**Affiliations:** Institute of Animal Reproduction and Food Research, Polish Academy of Sciences, Tuwima 10, 10-748 Olsztyn, Poland; d.rudewicz@pan.olsztyn.pl

**Keywords:** low-density lipoprotein (LDL), aptasensor, immunosensor, electrochemical detection

## Abstract

An elevated level of low density lipoprotein (LDL) can lead to the cardiovascular system-related diseases, such as atherosclerosis and others. Therefore, fast, simple, and accurate methods for LDL detection are very desirable. In this work, the parameters characterizing the electrochemical immuno-and aptasensor for detection of LDL have been compared for the first time. An immunosensor has been designed, for which the anti-apolipoprotein B-100 antibody was covalently attached to 4-aminothiophenol (4-ATP) on the surface of the gold electrode. In the case of an aptasensor, the gold electrode was modified in a mixture of ssDNA aptamer specific for LDL modified with –SH group and 6-mercaptohexanol. Square-wave voltammetry has been used for detection of LDL in PBS containing redox active marker, [Fe(CN)_6_]^3−/4−^. Our results show the linear dependence of [Fe(CN)_6_]^3−/4−^ redox signal changes on LDL concentration for both biosensors, in the range from 0.01 ng/mL to 1.0 ng/mL. The limit of detection was 0.31 and 0.25 ng/mL, for immuno- and aptasensor, respectively. Whereas slightly better selectivity toward human serum albumin (HSA), high density lipoprotein (HDL), and malondialdehyde modified low density lipoprotein (MDA-LDL) has been observed for aptasensor. Moreover, the other components of human blood serum samples did not influence aptasensor sensitivity.

## 1. Introduction

Low-density lipoproteins (LDL) transfer cholesterol and lipids from the liver to other organs within the plasma. They consist of triglycerides, cholesteryl esters, phospholipids, un-esterified cholesterol, and apolipoprotein B (apoB-100). These particles assume a spherical shape with an average diameter of 22 nm in which an amphipathic shell surrounds a hydrophobic lipid core. This outer shell comprises phospholipids, majority of un-esterified cholesterol and single copy of apoB-100. Depending on the lipid composition and the conformation of apoB-100, LDL particles adopt different structures and physical properties [1].

There is no doubt that LDL particles are the direct cause of atherosclerosis cardiovascular diseases (ASCVDs) [2,3]. The LDL level of ≥100 mg/dL is estimated to be a major risk for ASCVDs [4]. Moreover, the statistics show that 32% of deaths in the world are caused by these diseases (https://www.who.int/health-topics/cardiovascular-diseases accessed on: 11 June 2021). Thus, reliable and fast detection of ASCVDs biomarkers, such as LDL is extremely important in order to start treatment as early as possible [5].

In the laboratory practice, the measurement of LDL is based on the beta quantification procedure. The apoB lipoprotein particles are separated according to the hydrated density based on ultracentrifugation. This method has been established as a reference measurement procedure, but it is time-consuming, expensive, and requires special equipment [6]. Another method for LDL determination is based on quantitative measurements of total cholesterol, HDL, and triglycerides using the empirical relationship of Friedewald. This method is simple and cheap, but may show errors in calculation containing three parameters as well as limitations in using the Friedewald equation to the samples containing chylomicrons and concentration of plasma triglyceride over 400 mg/dL [7]. In many laboratories, precipitation and fully automated enzymatic method are used for the measurement of LDL [8].

The research interest in the electrochemical immunosensors has been constantly growing [9,10,11]. There is also a number of examples of electrochemical immunosensors for the LDL detection. In most of the presented literature examples, the authors have concentrated mainly on the nature and quality of the matrix utilized for immobilization of antibodies, in order to improve the biosensor performance. In a few examples, the deposition of apolipoprotein B-100 antibody has been reported on: NiO thin film [12], Langmuir-Blodgett films of polyaniline [13], aminated reduced graphene oxide modified electrode [14], L-cysteine in situ capped cadmium sulphide quantum dots bound to nickel oxide nanorods [15], carbon nanotubes, and chitosan composites deposited on an tin oxide-coated glass electrode [16]. These immunosensors have shown detection limits for LDL of 500 ng/mL [15], 5.0 × 10^4^ ng/mL [14], and 1.25 × 10^5^ ng/mL [16] calculated based on impedimetric response [14,16] or cyclic voltammetry [15].

The development of novel aptamers accelerates also the progress of electrochemical aptasensors [17,18,19]. Aptamers are oligonucleotides (short ssDNA or RNA) or peptides able to bind specifically appropriate molecule. Used as biological recognition element, they display many advantages compared to antibodies, mainly higher chemical stability, lower cost of production in chemical synthesis, and possibility of functional modifications [20]. To date, only one example of electrochemical aptasensor for detection of LDL has been reported [17]. The aptamer sequence applied in this research was first reported by Inapuri and co-workers in 2018 [21]. This 40-nucleotide ssDNA aptamer binds LDL with a 1.6 pM dissociation constant (kD) and weakly binds HDL and HSA. This sandwich-type aptasensor is based on aptamer-modified metal organic framework nanoparticles (MOF) and magnetic silica composite probe [17]. In the cited publication, an aptamer and ferrocene have been co-immobilized on MOF and used as a signal tag (MOF-Fc@Apt). While magnetic silica has been used as catcher of LDL (Fe_3_O_4_@SiO_2_-LDL). The created sandwich complex between MOF-Fc@Apt and Fe_3_O_4_@SiO_2_-LDL has been magnetically adsorbed on the surface of screen-printed electrode and detected electrochemically. This aptasensor could detect LDL in plasma in linear range of 1.0 ng/mL–100 µg/mL with the detection limit of 0.3 ng/mL (S/N = 3).

It is still under discussion which receptors, antibodies or aptamers, are better for selective, sensitive, repeatable, and low-cost electrochemical biosensor. There are not many literature examples for direct comparison between electrochemical immuno- and aptasensors. The example work has been presented by Prakash and co-workers [22]. The authors have conducted a comparative study for prostate specific antigen (PSA) detection using aptasensor and immunosensor based on graphene quantum dots-gold nanorods (GQDs-AuNRs)-modified carbon screen-printed electrodes. Under optimum conditions, both sensors have displayed comparable results and the same limit of detection of 0.14 ng/mL. However, in this study, bioreceptors, both antibodies and aptamers have been loaded on the modified surface of electrodes by adsorption leading to the un-oriented immobilization.

In the work presented here, the authors report a comparative study of immuno- and aptasensor for electrochemical detection of LDL for the first time. Both sensing platforms have been prepared by appropriate bioreceptors: antibodies or aptamers immobilization on the gold electrode surface via self-assembly processes. Apolipoprotein B monoclonal antibodies have been attached to—NH_2_ groups of 4-aminothiophenol previously deposited on gold. However, the accessibility of aptamer specific for LDL possessing—SH groups has allowed for its direct binding to the gold surface. The sensing of LDL has been done by square wave voltammetry experiments using [Fe(CN)_6_]^3−/4−^ as a redox marker in PBS.

## 2. Materials and Methods

### 2.1. Materials

Apolipoprotein B monoclonal antibody (AbM-anti-apoB) was purchased from Invitrogen (Poland). Aptamer specific to LDL antigen (Apt-LDL)—ssDNA sequence of

5′-ACCTCGATTTTATATTATTTCGCTTACCAACAACTGCAGA-3′ with 3′-SH group modification was synthesized by Biomers (Germany). 4-aminothiophenol (ATP), N-(3-Dimethylaminopropyl)-N′-ethylcarbodiimide hydrochloride (EDC), N-Hydroxysuccinimide (NHS), bovine serum albumin (BSA), phosphate buffer saline tablets: 0.01 M phosphate buffer, 0.0027 M potassium chloride, and 0.137 M sodium chloride, pH 7.4 at 25 °C (PBS), 6-mercapto-1-hexanol (6MHol), low density lipoprotein (LDL), human serum albumin (HSA), 4-mercaptobenzoic acid (4-MBA), K_2_Fe(CN)_6_, K_3_Fe(CN)_6_, were purchased from Sigma-Aldrich (Poznań, Poland). High density lipoprotein (HDL) was obtained From Merck (Germany). Malondialdehyde-modified low density lipoprotein (MDA-LDL) was acquired from Cell Biolabs, inc. (San Diego, CA, USA). H_2_SO_4_, KOH, ethanol, methanol were purchased from POCH (Poland). Alumina slurries: 0.3 and 0.05 µm were obtained from Buehler (Lake Bluff, IL, USA). Deionized water (resistivity of 18.2 MΩ cm) obtained with a Mili-Q reagent grade system produced by Millipore (Bedford, MA, USA) was used for preparation of all aqueous solutions. Human blood serum was obtained from Sigma-Aldrich. Mixed samples were aliquoted and stored at −20 °C until used.

### 2.2. Instruments

Electrochemical measurements were conducted with a potentiostat–galvanostat AutoLab (Metrohm Autolab, The Netherlands) with a three electrode configuration: gold as working electrode, Ag/AgCl as reference electrode, and Pt as counter electrode. Gold, reference, and platinum electrodes were manufactured by Bioanalytical Systems (BASi, West Lafayette, IN, USA).

### 2.3. Cleaning of Gold Electrode Surface

In the first step, Au electrodes were mechanically hand-polished with 0.3 and 0.05 µm alumina slurry for 5 min each. Then, the electrodes were electrochemically cleaned by conducting cyclic voltammetry (CV) in a solution of 0.5 M KOH and sweeping the potential between −1200 and −400 mV (vs. Ag/AgCl) at 100 mV/s scan rate during three, fifty, and five scans. After rinsing with Milli-Q water, electrodes were electrochemically cleaned in 0.5 M H_2_SO_4_ solution by changing the potential in CV from −300 to −1500 mV at 100 mV/s, during three, ten, and five scans. After another rinsing in Milli-Q water, the last ten scans of CV in 0.5 M KOH solution have been applied to Au electrodes, by changing the potential from −1200 to −400 mV at 100 mV/s. Such prepared electrodes, washed by Milli-Q water and dried in nitrogen, were ready for modification.

### 2.4. Preparation of Platform for

(a) immunosensor; initially, clean gold electrodes were immersed in an ethanolic solution of 1 mM 4-ATP overnight. Then, the electrodes were rinsed with ethanol and Milli-Q water to remove unbounded molecules. Afterwards, aqueous solution containing 0.05 mg/mL antibody (AbM-anti-apoB) and mixture of EDC/NHS (20 mM each) was incubated for 15 min at RT for activation of –COOH group present in AbM-anti-apoB. Next, 10 µL drop of an activated AbM-anti-apoB was deposited on the gold electrode for 2 h in RT. The unbounded AbM-anti-apoB molecules were rinsed with PBS. Then, the droplet of 1 mg/mL BSA solution in PBS was placed on the surface of gold electrode for 1 h followed by washing with PBS. The modified electrodes were kept overnight in PBS in 4 °C.
(b) aptasensor; To produce the aptasensor, first oligonucleotide aptamer (LDL-Apt) molecules were annealed by placing the sample in 90 °C for 10 min, followed by cooling down on ice for 15 min and in RT for 5 min. The 10 µL mixture of LDL-Apt (1.0 µM) and 6-MHol (10.0 µM) was dropped onto gold electrode surface and kept for 3 h in RT. Subsequently, electrodes were washed with PBS to remove any loosely bound aptamer molecules. Then, a drop of 1.0 mM 6-MHol solution in PBS was placed on electrode for another 30 min and again washed with PBS. Thus, the prepared electrodes were kept in PBS in refrigerator overnight.

### 2.5. Electrochemical Measurements of LDL

After overnight conditioning, both biosensors: immuno- and aptasensor were ready for electrochemical measurements. About 10 µL of sample solution containing particular concentration of LDL in PBS was dropped on the surface of modified gold electrodes. After 30 min of interaction between LDL and AbM-anti-apoB or Apt-LDL, electrodes were loaded in a PBS containing [Fe(CN)_6_]^3−/4−^ (1 mM) and square wave voltammograms were recorded in the range of −0.2 V to 0.6 V with 25 Hz frequency. In order to detect LDL in human serum samples, first the human blood serum samples were filtered with a Millipore AmiconUltracelYM-3, MWCO 3 kD and centrifuged for 60 min at 10,000 RCF in order to remove proteins with molecular weight over 3 kDa. The filtered human serum samples were used for preparation of LDL samples at the initial concentration of 1 mg/mL. The tested samples were then diluted with PBS to get final concentrations.

## 3. Results and Discussion

### 3.1. Optimization of Immunosensor Preparation

The appropriate orientation of antibody on the surface of electrode is crucial for specific non-covalent antibody-antigen interactions and as a consequence the electrochemical immunosensor efficiency. Many researchers have recently focused on the controllable and site-specific conjugation strategies of immunoglobulins immobilization on the surface of sensing platforms. These include, besides one step direct covalent immobilization, multi-step layer-mediated immobilization, such as protein A/G, Fc binding domain, biotin/(strept)avidin, SpyCather/SpyTag, etc., [23,24,25].

**Scheme 1:**
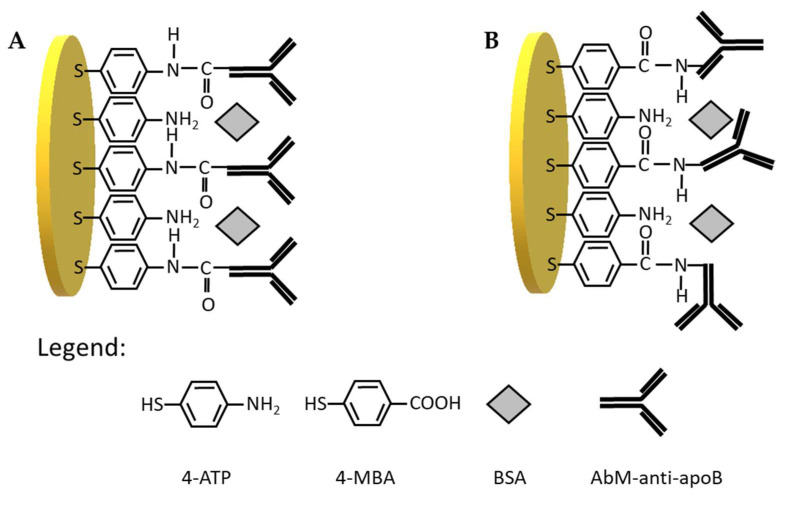
Schematic illustration of antibody immobilization on the gold electrode surface using (**A**) 4-ATP linker: Au/S(C_6_H_4_)NH-CO-AbM-anti-apoB/BSA and (**B**) 4-MBA linker: Au/S(C_6_H_4_)CO-NH-AbM-anti-apoB/BSA.

**Figure 1:**
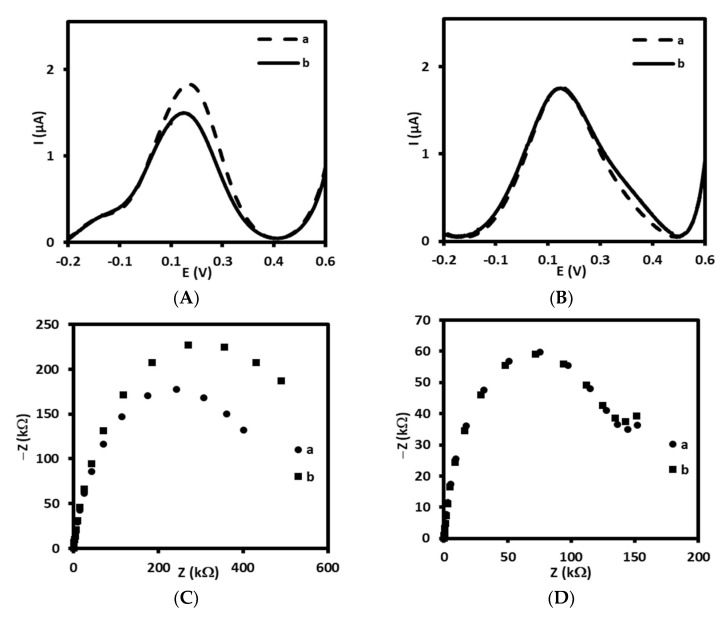
Square wave voltammograms and Nyquist plots recorded for immunosensor based on antibody immobilized on: (**A**,**C**) 4-ATP linker (a) before and (b) after interaction with 0.144 µM of LDL and (**B**,**D**) 4-MBA linker (a) before and (b) after interaction with 0.144 µM of LDL, recorded in PBS (pH 7.4) with 1 mM [Fe(CN)_6_]^3−/4−^.

Therefore, in the first step, we have tested two types of linkers for antibody immobilization on the gold electrodes, one containing –NH_2_ groups at the end, and other containing –COOH groups. Initially, 4-aminothiophenol was covalently immobilized on the surface gold electrode delivering free –NH_2_ groups available for covalent binding with-COOH groups from antibody (Scheme 1A). The receptor layer prepared in this manner has been treated with 0.144 µM LDL antigen. We have observed a decrease of current in square wave voltammetry by approximately 18% (Figure 1A, Table 1) and increase in the resistance of electrochemical impedance spectroscopy by 20% (Figure 1C, Table 1) upon interaction with LDL. These results have proved the efficient binding of LDL to antibody. Upon binding of LDL to appropriate antibody, the accessibility of [Fe(CN)_6_]^3−/4−^ to the surface of gold electrode was decreased leading to the diminution of its reduction/oxidation current. On the other hand, we have also tested 4-mercaptobenzoic acid possessing –COOH groups as linker for antibody immobilization (Scheme 1B). In this case, we have not observed any changes in SWV and EIS upon the interaction with LDL (Figure 1B,D, Table 1). Probably, the reason is the inappropriate orientation of antibody on the surface of the gold electrode. The influence of antibody attachment to the phenyl films containing either –COOH or –NH_2_ groups on the efficiency of antibody ferritin binding has been reported by Matysiak-Brynda and co-workers. The authors have observed that the vertical orientation of antibody vs the electrode surface could be achieved by its binding to aminoethylophenyl linkers leading to the increasing efficiency of sensor toward electrochemical detection of ferritin [26].

**Scheme 2:**
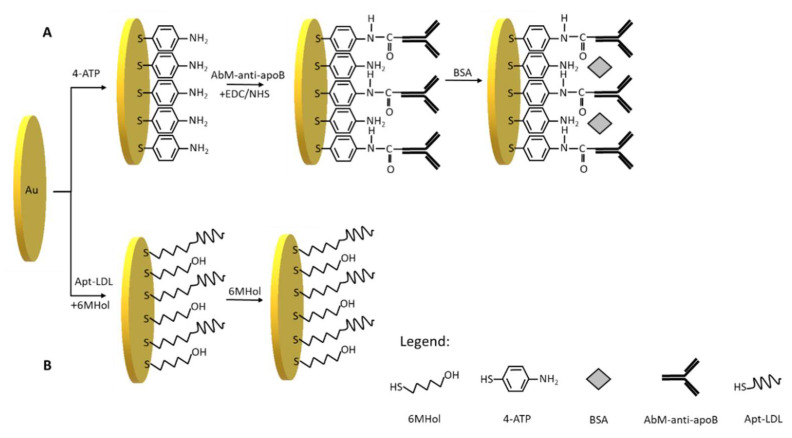
Schematic illustration of steps for the fabrication of (**A**) the immunosensor: Au/4-ATP/AbM-anti-apoB/BSA and (**B**) the aptasensor: Au/Apt-LDL,6MHol.

Therefore, we have used 4-aminothiophenol as a linker in the procedure of immunosensor preparation (Scheme 2A). This linker has been successfully applied in a few electrochemical immunosensors [27,28,29]. After 4-ATP covalent immobilization on the gold electrode surface, the activated by EDC/NHS AbM-anti-apoB antibody has been covalently attached to the electrode. The incubation of electrode with a BSA solution in the next step prevents non-specific interactions. The platform designed in this way is based on a 3-steps modification, the preparation of which takes 22 h.

### 3.2. Optimization of Aptasensor Preparation

In this work, we have applied the procedure of aptasensor preparation developed earlier in our laboratory [30]. In this optimized procedure (Scheme 2B), the first step is to modify the gold electrodes with ssDNA aptamer specific for LDL (Apt-LDL) in the mixture of 6-mercapto-1-hexanol in the concentrations: 10^−6^ and 10^−5^ M, respectively. The next step of aptasensor preparation is based on self-assembling of 6-MHol in order to block non-modified Au area and avoid non-specific interactions. Such procedure has been widely applied in the preparation of many sensors [31,32,33]. This 2-step modification takes 3.5 h to prepare.

### 3.3. Electrochemical Characterization of Immuno- and Aptasensor

**Figure 2:**
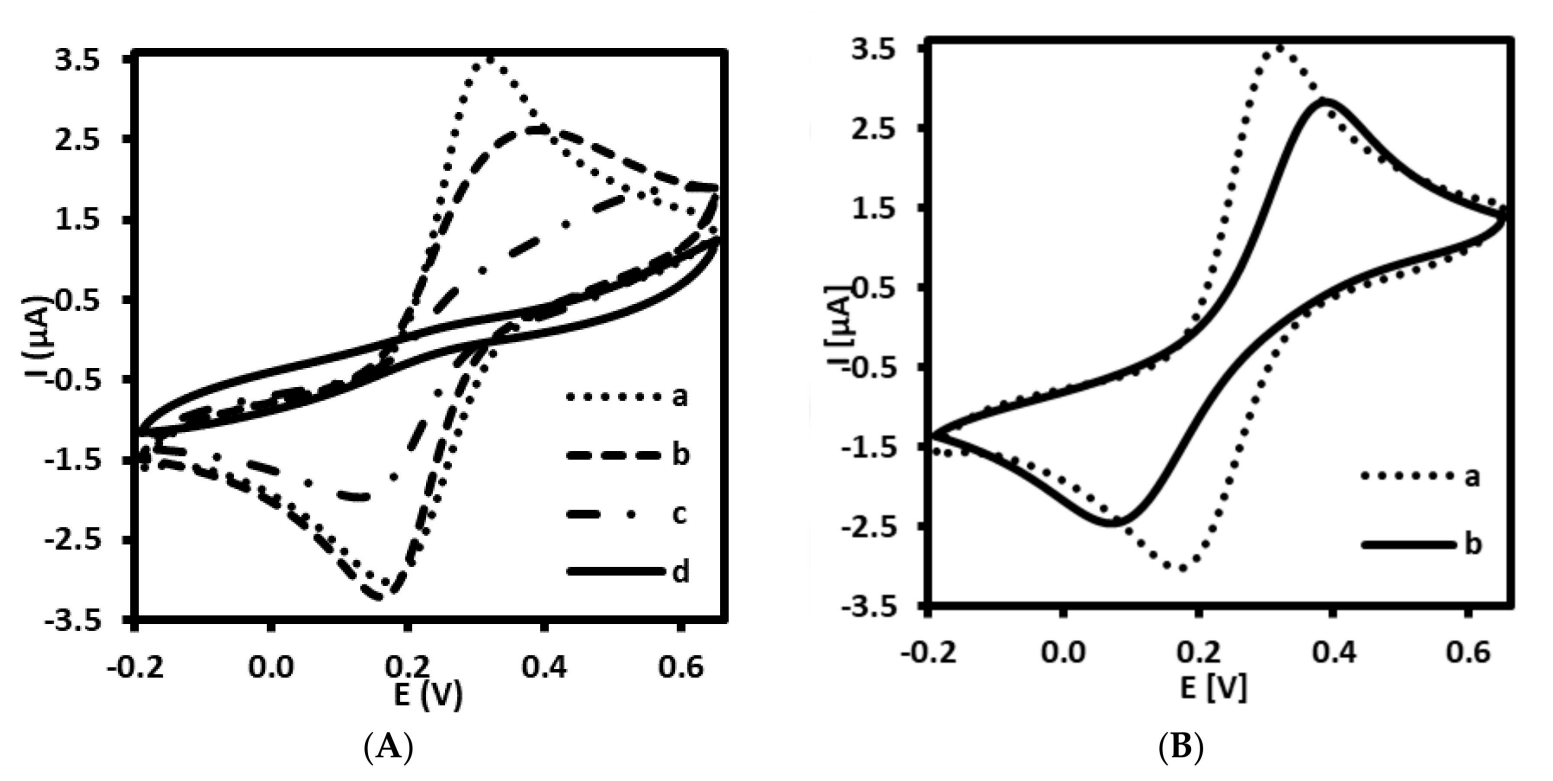
Cyclic voltammograms recorded for (**A**) immunosensor at different stages of fabrication: (a) bare gold electrode (Au), (b) Au/4-ATP, (c) Au/4-ATP/AbM-anti-apoB, (d) Au/4-ATP/AbM-anti-apoB/BSA, and (**B**) aptasensor at different stages: (a) bare gold electrode (Au), (b) Au/LDL-Apt, 6MHol. Supporting electrolyte: PBS (pH 7.4) with 1 mM [Fe(CN)_6_]^3−/4−^.

Each step of the immuno- and aptasensor fabrication processes has been characterized by the recording cyclic voltammetry measurements that indicate the changes in charge transfer of redox active pair [Fe(CN)_6_]^3−/4−^. As shown in Figure 2A,B, the bare Au electrode (curve a) exhibits a reversible behavior toward redox forms present in the electrolyte solution. The CV performed at 100 mV/s showed two peaks characterized by peak separation (ΔE) of 85.0 ± 2.9 mV (*n* = 5). The pair of peaks characteristic of ferri/ferrocyanide has gradually decreased after immobilization of 4-ATP (Figure 2A, curve b) and their ΔE was equal to 198.0 ± 39.6 mV (*n* = 5). Similar phenomena has been observed by other authors [27,28,29]. The deposition of 4-ATP on the Au electrode surface caused the decrease of Fe(CN)_6_]^3−/4−^ redox current. Indeed, taking into account the presence of positively charged amino ends of 4-ATP (predicted pK_a_= 8.74 ± 0.10), we could expect the promotion of electron transfer between anionic ferri/ferrocyanide and protonated amine groups at pH 7.4, as observed for example on self-assembled 6-amino-1-hexanethiol monolayer on gold [34]. Probably, the insulating properties of densely packed self-assembled layer of 4-ATP (modification conditions: 1 mM of 4-ATP, overnight) have a greater inhibitory effect on the electron transfer of anionic redox marker ([Fe(CN)_6_]^3−/4−^) than the promoting effect of electrostatic interaction between positively charged monolayer and anionic marker. Another reduction of the peak current was observed after attachment of AbM-anti-apoB (Figure 2A, curve c) and final filling with BSA (Figure 2A, curve d).

Furthermore, CV has been also adapted in order to prove the fabrication process of the aptasensor. Similar tendency has been observed for aptasensor as compared with immunosensor. The self-assembling of SH-ssDNA aptamer probe together with 6-MHol has also led to blocking effect of this receptor and filler on interfacial electron transfer of redox probe (Figure 2B, curve b). The peak separation observed for modified gold electrode was 252.0 ± 4.0 mV (*n* = 5).

**Figure 3:**
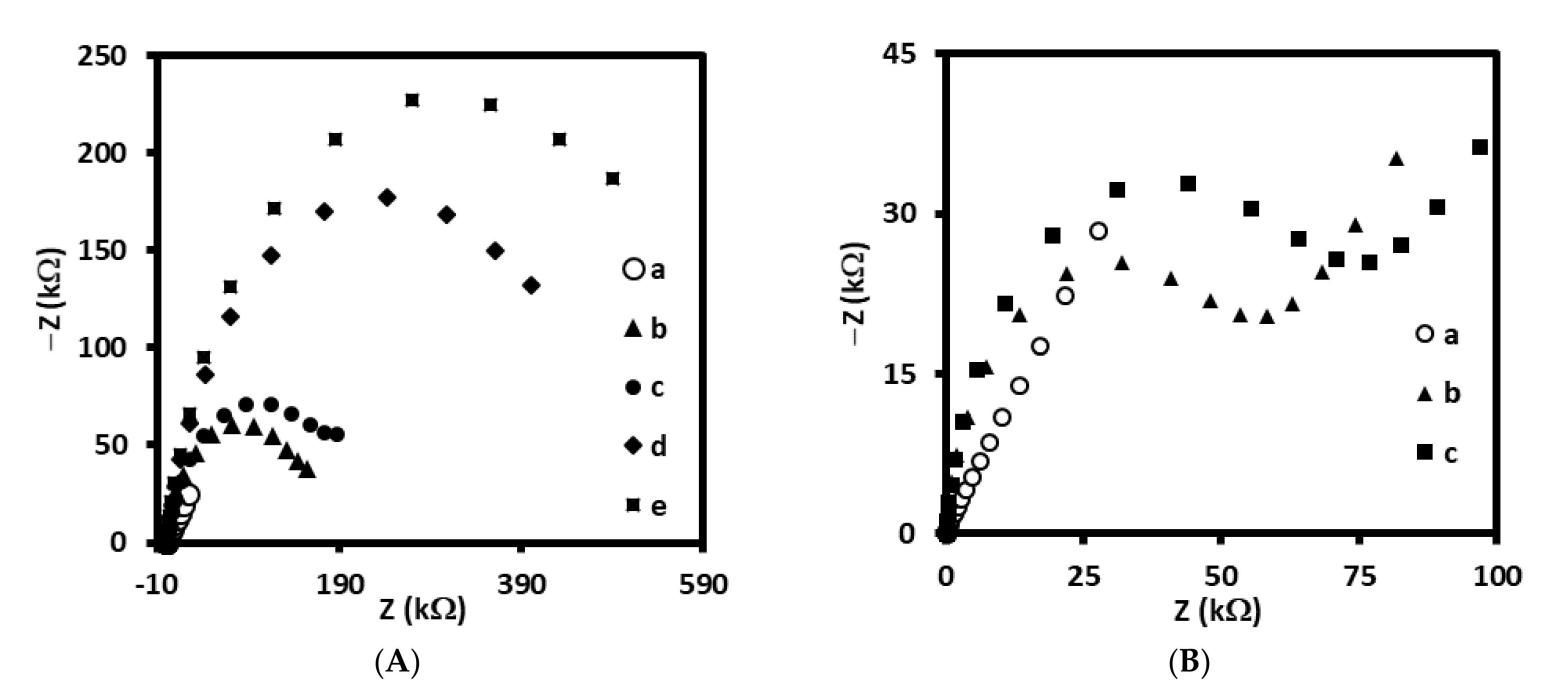
Impedance spectroscopy recorded for (**A**) immunosensor at different stages of fabrication: (a) bare gold electrode (Au), (b) Au/4-ATP, (c) Au/4-ATP/AbM-anti-apoB, (d) Au/4-ATP/AbM-anti-apoB/BSA, (e) Au/4-ATP/AbM-anti-apoB/BSA/LDL, and (**B**) aptasensor at different stages: (a) bare gold electrode (Au), (b) Au/LDL-Apt, 6MHol, (c) Au/LDL-Apt, 6MHol/LDL. Supporting electrolyte: PBS (pH 7.4) with 1 mM [Fe(CN)_6_]^3−/4−^.

Additionally, each modification step for both immuno- and aptasensor has been confirmed by impedance spectroscopy. The increase of resistance of electrodes for each step has been observed. In addition, an interaction between monoclonal antibody and LDL or aptamer and LDL has been proved and the increase of resistance has been observed (Figure 3A,B).

These results have proved the individual stages of gold electrodes modification with specific receptors, both antibodies and aptamers.

### 3.4. Quantitative Electrochemical Detection of LDL by Immuno- and Aptasensor

**Figure 4:**
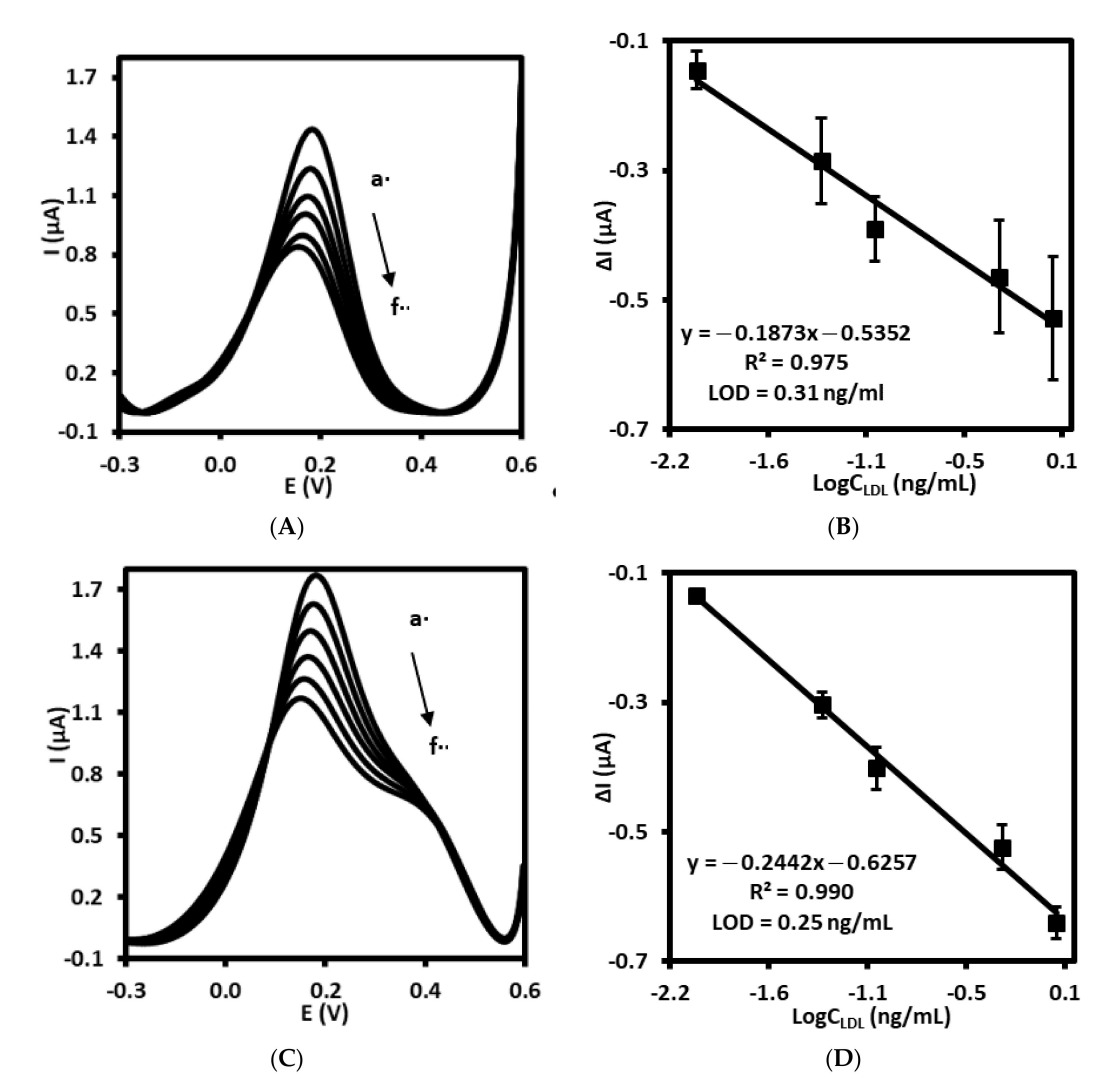
Square wave voltammograms recorded for (**A**) immunosensor and (**C**) aptasensor upon treatment with LDL standard solution with different concentration, a: 0.0, b: 0.01, c: 0.05, d: 0.1, e: 0.5, f: 1.0 ng/mL in PBS (pH 7.4) with 1 mM [Fe(CN)_6_]^3−/4−^. The calibration curves of (**B**) immunosensor and (**D**) aptasensor for LDL detection.

The immunosensor prepared by gold electrode modified with 4-ATP, followed by AbM-anti-apoB and BSA (Au/4-ATP/AbM-anti-apoB/BSA) has been used for electrochemical detection of LDL by square wave voltammetry recorded in PBS containing 1 mM [Fe(CN)_6_]^3−/4−^, pH 7.4. The oxidation/reduction current of ferri/ferrocyanide recorded using electrode modified with AbM-anti-apoB in PBS free of LDL was in the range 1.2 ± 0.2 µA. The position of peak current was equal to 147 ± 4.4 mV (*n* = 3). As presented in Figure 4A, maximum peak current has gradually decreased with increase of LDL concentration in the range from 0.01 to 1.0 ng/mL. With the increasing concentration of LDL, the number of AbM-anti-apoB–LDL immuno-complexes is increasing. Moreover, the isoelectric point of LDL is 5.2 [35], so, this lipoprotein is negatively charged at pH 7.4. Thus, the electrostatic repulsion between negatively charged LDL and negatively charged [Fe(CN)_6_]^3−/4−^ redox couple occurs hence blocking redox reaction and hindering the electron transfer. The sensing mechanism has been illustrated and described in more details and in Supplementary materials. Respectively, the linear relationship between Δcurrent and logarithm of LDL concentration has been observed, characterized by regression equation: Δcurrent = −0.1873logC_LDL_ − 0.5352, the correlation coefficient of 0.975 (R^2^) and limit of detection (LOD) of 0.31 ng/mL (S/N = 3.3) (Figure 4B).

Similarly, the gold electrode modified with ssDNA-LDL aptamer together with 6MHol (Au/LDL-Apt, 6MHol) has been tested as aptasensor for electrochemical LDL detection. For aptasensor, the peak current of [Fe(CN)_6_]^3−/4−^ measured in PBS with SWV was in the range of 1.7 ± 0.1 µA and the position of 151.0 ± 4.3 mV (*n* = 5). The same phenomenon has been observed as compared with immunosensor. The interaction of ssDNA aptamer with LDL as its concentration increased has led to the lowering of the peak current of the redox pair ferri/ferrocyanide present in the supporting solution (Figure 4C). The linear relationship between Δcurrent and log concentration of LDL has been observed in the concentration range between 0.01 and 1.0 ng/mL with a regression equation: Δcurrent = −0.2442logC_LDL_ − 0.6257, correlation coefficient R^2^ of 0.990. The calculated limit of detection for this aptasensor was 0.25 ng/mL(S/N = 3.3) (Figure 4D). Thus, both sensors, immuno- and aptasensor have displayed very similar analytical parameters, including a detection limit.

Table 2 presents selected examples of electrochemical immunosensors and one example of aptasensor for LDL published so far. The comparison of immunosensors presented with the immunosensor described in this work has led to the following conclusions: most of the examples have been characterized by LOD in the range of more than hundred ng/mL of LDL [14,15,16,36,37,38]. In another example presented by Yan and co-workers, a hybrid material composed of silver chloride@polyaniline core-shell (AgCl@PANI) combined with Au nanoparticles (AuNPs)—AuNPs-AgCl@PANI modified glassy carbon electrode has been used for adsorption of antibody anti apolipoprotein B-100 [39]. A much better detection limit of 0.34 × 10^−3^ ng/mL as compared to the other examples has been obtained in this case using electrochemical impedance spectroscopy as a measuring technique. Indeed, the authors have obtained the ultralow detection limit, probably due to the large surface of volume ratio of AuNPs-AgCl@PANI leading to high antibody loading. However, they did not tested this immunosensor in real samples. Whereas, the only electrochemical aptasensor for the detection of LDL reported so far has been characterized by a very similar detection limit of 0.3 ng/mL as compared to our aptasensor [17]. Somehow, the sandwich-type laborious construction of this aptasensor demands conjugation of LDL on Fe_3_O_4_@SiO_2_ and immobilization of aptamer and ferrocene on UiO-66 metal organic framework. This slightly reduced the probability of its application for point of care testing of LDL. Moreover, our system is characterized by an easy and rapid experimental protocol, reasonable cost, and no need for the use of expensive nanomaterials.

### 3.5. Specificity and Repeatability of Immuno- and Aptasensor

**Figure 5:**
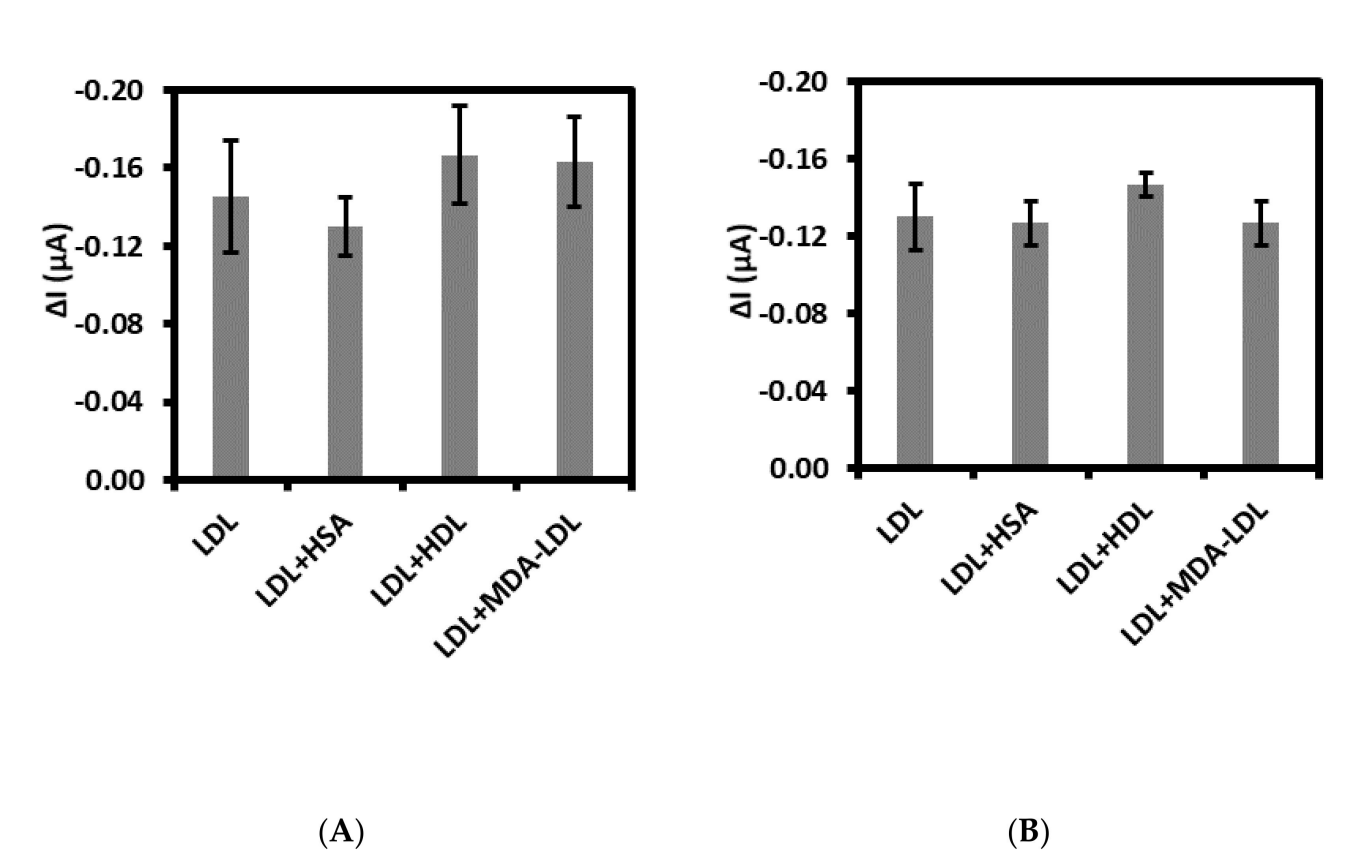
Selectivity of (**A**) immunosensor and (**B**) aptasensor with 0.01 ng/mL LDL along with different interferents: HSA, HDL, and MDA-LDL at 0.5 ng/mL concentration each.

Specificity defined as the ability to detect specifically and accurately LDL in the presence of other interferents possibly present in the sample has been evaluated [40]. For this purpose, both immuno- and aptasensor were controlled for the detection of LDL at the concentration of 0.01 ng/mL along with 50-fold higher concentration of possible interferents. We have tested the human plasma proteins as follows: human serum albumin, high density lipoprotein, and malondialdehyde-modified low density lipoprotein. As shown in Figure 5A,B, HSA did not have big influence on the detection of LDL in the case of both sensors. Whereas the small increase of Δcurrent for HDL has been observed, about 15% and 13% for immuno- and aptasensor, respectively. While the slight increase of Δcurrent for MDA-LDL in comparison with LDL has been observed for immunosensor, equal to 12% (Figure 5A). No influence of MDA-LDL on LDL detection has been observed for aptasensor. Moreover, the acceptable relative standard deviations (RSDs) has been calculated for aptasensor (HSA: 3.4%, HDL: 4.2%, MDA-LDL: 6.9%). So, these results confirm the high selectivity of aptasensor toward LDL. Especially, the lack of interferences of MDA-LDL on the detection of LDL is very promising. Since, the MDA-modified LDL-systemic oxidative stress biomarker is a very close analogue of LDL, differing in the structure of LDL by MDA bounded to positively charged epsilon-amino group of apo B-100 protein lysyl residues [41].

Moreover, the repeatability, referred to as ability to generate the same results during the short time under the same conditions (intra-assay precision) [40] has also been evaluated for immuno- and aptasensor. Our data demonstrated that repeatability of the aptasensor proposed for LDL detection was acceptable in the range between 3.1% and 5.9% and clearly better than analogous immunosensor.

**Figure 6:**
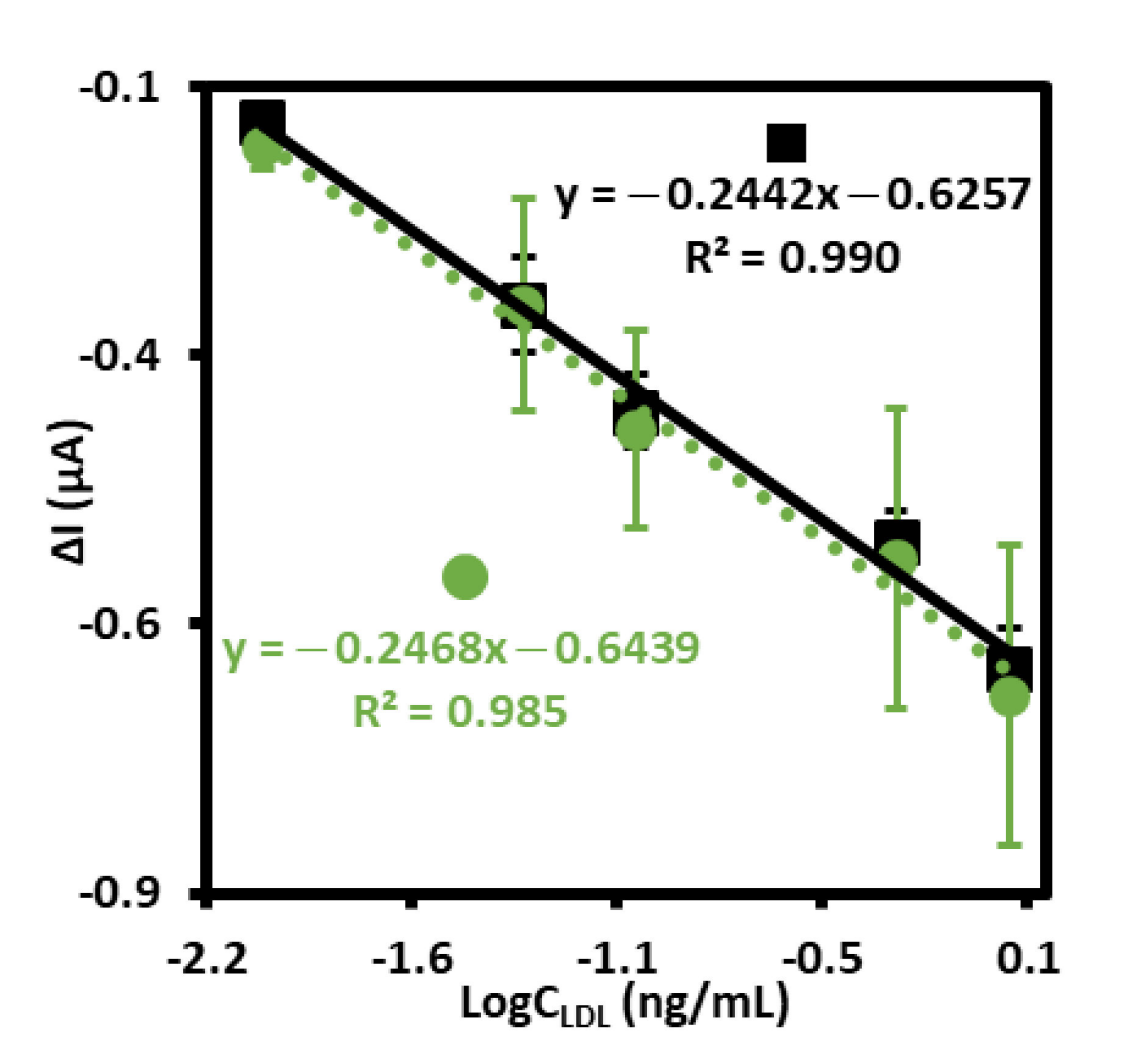
The calibration curves of aptasensor to LDL standard solution with different concentrations (0.01–1.0 ng/mL) (■ solid line), and to LDL standard solution with different concentrations (0.01–1.0 ng/mL) prepared with human serum sample after filtration on Amicon Ultra Centrifugal Filter 3 kDa (•dotted line), both recorded in PBS (pH 7.4) with 1 mM [Fe(CN)_6_]^3−/4−^.

The positive results of specificity tests, repeatability, as well as parameters of sensor preparation: shorter time of modification, price of aptamer synthesis, and availability of the –SH group-modified aptamer allowed us for further testing this electrochemical aptasensor for detection of LDL. In order to check if other components of human serum samples, in dimension smaller than 3 kD influence LDL detection, the following experiment has been conducted. First, the samples of human serum were centrifuged for 60 min at 10,000 RCF with a Millipore Amicon Ultracell YM-3. Then, the filtered plasma was used for the preparation of LDL at the concentration of 1 mg/mL and finally diluted to the final concentration of 0.01, 0.05, 0.1, 0.5, 1.0 ng/mL with a PBS. Finally, the calibration curve has been prepared and compared with standard calibration curve as illustrated in Figure 6. The calculated limit of detection, LOD = 0.29 ng/mL (S/N = 3.3) has been comparable to the LOD obtained for standard calibration curve (0.25 ng/mL, S/N = 3.3). Very similar regression coefficients for both curves have also been obtained and equaled 0.985 and 0.990 for the experiment conducted in human serum sample and PBS, respectively. Almost the same slopes have also been obtained for both curves and equaled −0.2442 and −0.2468 for standard calibration curve and human serum sample, respectively. When comparing the results received in both experiments, it should be assumed that the electrochemical aptasensor for detection of LDL could possibly find practical application.

## 4. Conclusions

In summary, an electrochemical immuno- and aptasensor for detection of low density lipoprotein have been compared for the first time. Herein, the oriented immobilization of monoclonal antibody (AbM-anti-apoB) or aptamer (Apt possessing –SH group) on the surface of gold electrodes has been designed. As a result of the formation receptor—analyte complexes, precisely AbM-anti-apoB or Apt and negatively charged LDL analyte at pH 7.4, the repulsion of [Fe(CN)_6_]^3−/4−^ redox probe from the electrode surface occurred. The decrease in electron transfer and lowering the peak current in square wave voltammetry used as measuring technique has been observed. Both sensors have displayed linear relationship between Δcurrent and log concentration of LDL in the concentration range between 0.01 and 1.0 ng/mL and almost the same limit of detection equal to 0.31 ng/mL and 0.25 ng/mL for immuno- and aptasensor, respectively. The LOD obtained for electrochemical immunosensor is superior in comparison with the most presented so far immunosensors. Our results show that electrochemical aptasensor based on modification of gold electrode with LDL specific ssDNA aptamer having –SH group, together with 6-mercapto-1-hexanol is sufficient to detect electrochemically LDL in the human serum samples with the low detection limit of 0.25 ng/mL. However, LOD obtained in this work is similar to single example of biosensor utilizing advanced electrode materials, complicated and prolonged procedures of sensor fabrication. The satisfactory specificity of aptasensor has been also observed. The lack of interferences from the main serum proteins, specifically human serum albumin, high density lipoprotein, and malondialdehyde-modified LDL was observed. Due to the ultralow detection limit and the dilution of the sample, slight interferences from natural components present in the human serum samples has also been observed. We do believe that the developed aptasensor could find practical application in laboratory diagnostic in the future.

## Figures and Tables

**Table 1:** The values of changes in current (ΔI, ΔI/I) and changes in resistance (ΔR, ΔR/R) registered for electrodes modified with AbM-anti-apoB antibody and 4-ATP linker (Au/S(C_6_H_4_)NH-CO-AbM-anti-apoB/BSA) or 4-MBA linker (Au/S(C_6_H_4_)CO-NH-AbM-anti-apoB/BSA).

	ΔI (µA)	SD	ΔR (kΩ)	SD	ΔI/I (%)	SD	ΔR/R (%)	SD
Au/S(C6H4)NH-CO-AbM/BSA/LDL	−0.34	0.02	105	19.7	−18.1	1.40	20.2	5.72
Au/S(C6H4)CO-NH-AbM/BSA/LDL	−0.10	0.13	−1.17	5.97	−4.59	5.73	−0.53	5.68

ΔI = I_n_ − I, which means: I_n_—value of the peak current measured in the presence of particular “n” concentration of LDL, recorded at the potential of maximum peak current value registered in buffer, I—value of peak current measured in a solution free of LDL, in buffer.

**Table 2:** Comparison of various biosensors for the detection of LDL described in the literature with the immunosensor (Au/4-ATP/AbM-anti-apoB/BSA) and aptasensor (Au/Apt-LDL, 6MHol) developed in this work.

Substrate	Receptor	Electrochemical Method	Limit of Detection ng/mL	Ref.
AuNPs-AgCl@PANI	Antibody	EIS	0.34 × 10^−3^	[39]
CysCdS-nNiO/ITO	Antibody	CV	500	[15]
CNT−NiO	Antibody	EIS	6.3 × 10^3^	[37]
rGO-NiO	Antibody	EIS	700	[38]
CNT-CH/ITO	Antibody	EIS	1.25 × 10^5^	[16]
Polyaniline	Antibody	EIS	-	[13]
NiO	Antibody	EIS	-	[12]
NH_2_-rGO/ITO	Antibody	EIS	5.0 × 10^4^	[14]
CysCdS/Au	Antibody	EIS	16.03 × 10^4^	[36]
Fe_3_O_4_@SiO_2_ and MOF-Fc@APT	Aptamer	SWV	0.3	[17]
Au/4-ATP/AbM/BSA	Antibody	SWV	0.31	Present work
Au/Apt	Aptamer	SWV	0.25	

AuNPs—Au nanoparticles, AgCl@PANI—silver chloride@polyaniline, CysCdS-nNiO–L—cysteine capped cadmium sulphide quantum dots bound to nickel oxide nanorods, ITO –indium tin oxide, CNT—carbon nanotubes, rGO—reduced graphene oxide, CH—chitosan, NH_2_-rGO—aminated reduced graphene oxide, Fe_3_O_4_@SiO_2_—silica coated magnetite nanoparticles, MOF-Fc –ferrocene immobilized metal organic framework, APT—aptamer, 4-ATP—4—aminothiophenol, AbM—monoclonal antibody, BSA—bovine serum albumin, EIS—electrochemical impedance spectroscopy, SWV—square wave voltammetry.

## Data Availability

The data presented in this study are available on request from the corresponding author.

## Supplementary Materials

The following illustration of sensing mechanism are available online at https://www.mdpi.com/article/10.3390/s21227733/s1. Figure S1: the ilustration of sensing mechanism.
