# Supplementary material for: Detection of Low Density Lipoprotein—Comparison of Electrochemical Immuno- and Aptasensor
Source: Sensors (Basel). 2021 Nov 20;21(22):7733. doi: 10.3390/s21227733 (PMC8620298; doi:10.3390/s21227733)
Supplement: Supplementary file 1 [file sensors-21-07733-s001.zip › sensors-1459421-supplementary.pdf]

Supplementary Materials

Detection of low density lipoprotein - comparison of electrochemical immuno- and aptasensor

Daria Rudewicz-Kowalczyk[1], Iwona Grabowska[1]*

* **Correspondence: i.grabowska@pan.olsztyn.pl**

In this approach, changes in electrochemical parameters of the interface electrode/aqueous solution caused by antibody-antigen and aptamer-analyte event have been monitored. In this type of biosensors, the mechanism of the signal generation, originally developed by Umezawa, is related to an ion-channel mimetic system (**Umezawa Y, Aoki H (2004) Ion channel sensors based on artificial receptors. Anal Chem 76: 320A–326A**). Here, upon the binding of LDL to the biological receptors: antibody or aptamer immobilized on the electrode surface, the access of anionic ($[Fe(CN)_6]^{3-/4-}$) marker ions present in the sample solution to the modified surface is decreased. With the increasing concentration of LDL, the number of AbM-anti-apoB – LDL immuno-complexes is increasing. Moreover, the isoelectric point of LDL is 5.2, so, at pH 7.4, this lipoprotein is negatively charged. Thus, the electrostatic repulsion between negatively charged LDL and negatively charged $[Fe(CN)_6]^{3-/4-}$ redox couple occurs hence blocking redox reaction and hindering the electron transfer.

A scheme of the working principle of the ion-channel mimetic sensor, where anionic marker ions exist in the sample solution is shown below.

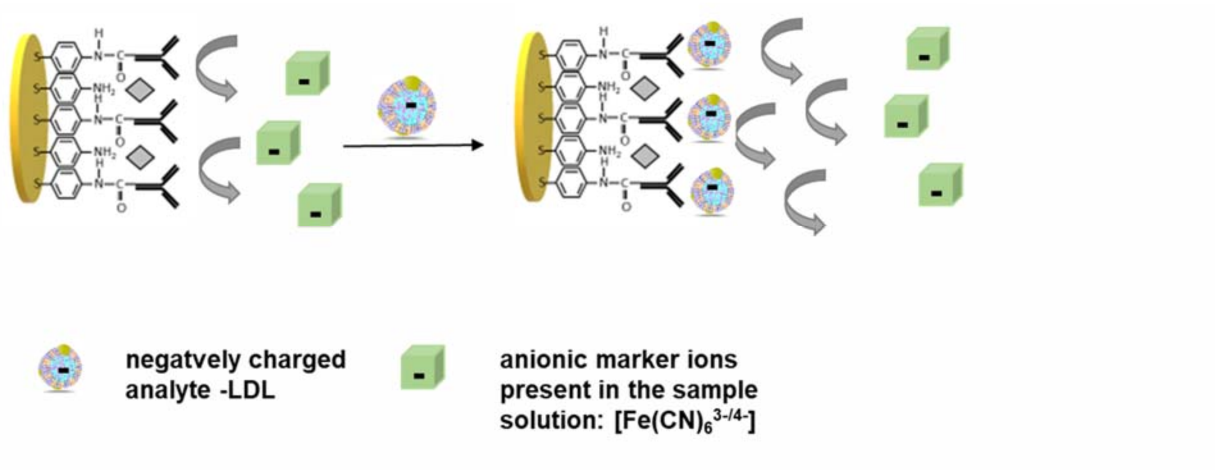

Figure S1: the ilustration of sensing mechanism.
